# The accessory proteins REEP5 and REEP6 refine CXCR1-mediated cellular responses and lung cancer progression

**DOI:** 10.1038/srep39041

**Published:** 2016-12-14

**Authors:** Cho Rong Park, Dong-Joo You, Sumi Park, Sunam Mander, Da-Eun Jang, Su-Cheong Yeom, Seong-Hyun Oh, Curie Ahn, Sang Heon Lee, Jae Young Seong, Jong-Ik Hwang

**Affiliations:** 1 Graduate School of Medicine, Korea University, Seongbuk-gu, Seoul 02841, Republic of Korea; 2 Graduate School of International Agricultural Technology, Seoul National University, 1447 Pyeongchang-Ro, Daewha, Pyeongchang, Kangwon, 25354, Republic of Korea; 3 College of Pharmacy, Gachon University, Incheon, 406-840, Republic of Korea; 4 Transplantation Research Institute, Cancer Research Institute, Seoul National University, Yongun-dong, Jongno-gu, Seoul 110-799, Republic of Korea; 5 Department of Physical Medicine and rehabilitation, Korea University, Seongbuk-gu, Seoul 02841, Republic of Korea

## Abstract

Some G-protein-coupled receptors have been reported to require accessory proteins with specificity for proper functional expression. In this study, we found that CXCR1 interacted with REEP5 and REEP6, but CXCR2 did not. Overexpression of REEP5 and REEP6 enhanced IL-8-stimulated cellular responses through CXCR1, whereas depletion of the proteins led to the downregulation of the responses. Although REEPs enhanced the expression of a subset of GPCRs, in the absence of REEP5 and REEP6, CXCR1 was expressed in the plasma membrane, but receptor internalization and intracellular clustering of β-arrestin2 following IL-8 treatment were impaired, suggesting that REEP5 and REEP6 might be involved in the ligand-stimulated endocytosis of CXCR1 rather than membrane expression, which resulted in strong cellular responses. In A549 lung cancer cells, which endogenously express CXCR1, the depletion of REEP5 and REEP6 significantly reduced growth and invasion by downregulating IL-8-stimulated ERK phosphorylation, actin polymerization and the expression of genes related to metastasis. Furthermore, an *in vivo* xenograft model showed that proliferation and metastasis of A549 cells lacking REEP5 and REEP6 were markedly decreased compared to the control group. Thus, REEP5 and REEP6 could be novel regulators of G-protein-coupled receptor signaling whose functional mechanisms differ from other accessory proteins.

G protein-coupled receptors (GPCRs) are cell surface proteins that bind to extracellular ligands, activate intracellular signaling pathways, and play key roles in neurotransmission, secretion, muscle contraction, blood pressure regulation, immune response, and cancer progression^1^,^2^,^3^. GPCR-mediated signaling events are regulated by GPCR kinases (GRKs), arrestins, and regulators of G protein signaling (RGS). RGS promotes GTP hydrolysis via the α subunit of heterotrimeric G proteins, thereby switching off GPCR signaling pathways^4^. After G protein-binding and release, the GPCR intracellular domains undergo phosphorylation by GRKs or protein kinase A and C^5^,^6^, which provides binding sites for β-arrestins that block further G protein-mediated signaling and target the receptors for internalization^7^,^8^. Although the primary roles of β-arrestins are downregulation of GPCR signaling, they also stimulate diverse intracellular signaling pathways. However, these canonical or alternative signaling events do not explain the tissue- or cell-specific responses mediated by GPCRs in response to extracellular stimuli.

Some chaperone proteins, including Nina A, mediate cell surface expression of GPCRs^9^,^10^, whereas others, including calnexin, assist with the glycosylation and folding of newly synthesized membrane proteins or target improperly folded molecules for degradation^11^,^12^. Three subtypes of receptor activity modifying proteins (RAMPs) lead to different receptor glycosylation states and confer receptor-binding selectivity for adrenomedullin versus calcitonin gene-related peptides^13^, which implies that specific accessory proteins expressed with tissue- or cell type-specificity could determine the functional maturity of some GPCRs. According to recent studies, odorant receptors in olfactory neurons require accessory proteins, including receptor expressing enhancing protein 1 (REEP1), for proper cell surface expression^14^. Although most functional roles of these proteins have been related to neuronal cell-specific GPCRs, some are expressed in other cells or tissues, suggesting that they might regulate proper expression and function of many GPCRs or other membrane proteins.

In this study, we screened chemokine receptors affected by accessory proteins and demonstrated that REEP5 and REEP6 enhanced interleukin-8 (IL-8)-stimulated CXCR1 activation. The effects of REEPs on CXCR1-related cellular responses were analyzed in heterologous cells and lung cancer cells expressing endogenous CXCR1. The results provided evidence of the functional roles of accessory proteins in cellular systems other than neurons and enhanced the understanding of the regulation of REEPs in chemokine signaling.

## Results

### CXCR1-mediated signaling is enhanced by exogenous REEP5 and REEP6

**Figure 1:**
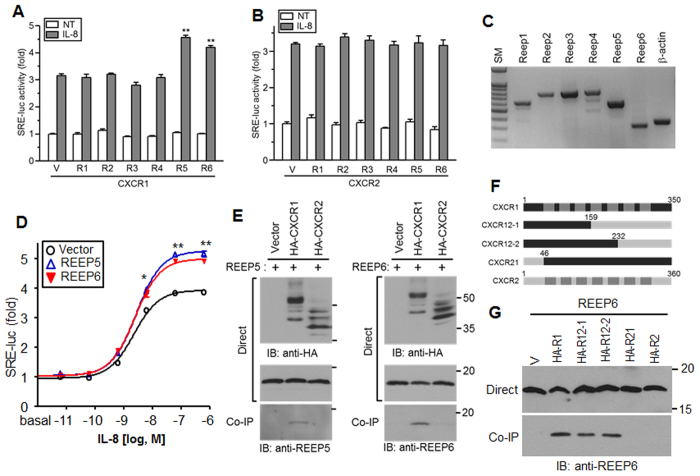
Expression of REEP5 and REEP6 enhances IL-8 receptor CXCR1-mediated signaling. (**A**) HEK293/Gα_15_ cells transfected with the *SRE*-*luc* reporter, CXCR1 plasmids, and *REEP* were treated with 10 nM IL-8 and assayed for luciferase activity. **p < 0.01. NT; no treatment, V; vector, R; REEP. (**B**) Luciferase activity was assayed using cells expressing CXCR2. (**C**) Total RNA was extracted from HEK293 cells and expression of *REEP* was determined by RT-PCR. The expected PCR product sizes are indicated by an asterisk. (**D**) HEK293 cells transfected with *SRE*-*luc, CXCR1*, and either *REEP5* or *REEP6* were treated with 10-fold serially diluted IL-8 (from 602 nM) and assayed for luciferase activity. *p < 0.05, **p < 0.01. (**E**) Lysates from HEK293 cells transfected with HA-CXCR1 and REEP5 or REEP6, or HA-CXCR2 and REEP5 or REEP6 were applied for immunoprecipitation with anti-HA agarose and analyzed by western blotting with relevant antibodies. (**F**) Schematic representation of CXCR1 and CXCR2. Shaded regions represent the transmembrane domains. The chimeric receptors were generated by PCR. The designated numbers are the chimeric position. Dark gray indicates the transmembrane domain. (**G**) A co-immunoprecipitation assay was performed with the HA-tagged chimeric CXCR1/2 receptors and REEP6, and analyzed by western blotting with anti-REEP6 antibodies. HA-R, HA-CXCR. All experiments were performed at least three times.

To explore the effects of REEPs on IL-8-stimulated cellular responses, *CXCR1* and *CXCR2* were transfected with *REEP* and the *SRE*-*luc* reporter into HEK293 cells harboring Gα_15_. IL-8 treatment caused reporter gene expression, which was mediated by Gα_15_ and downstream protein kinase C activation in cells expressing CXCR1 and CXCR2. Interestingly, luciferase activity was enhanced in cells transfected with *CXCR1* and *REEP5* or *REEP6* (Fig. 1A), but not in cells expressing CXCR2 (Fig. 1B), which implied that REEP5 and REEP6 likely affected CXCR1-mediated signaling with receptor type-specificity.

We examined the expression of *REEP* in HEK293 cells by reverse transcription-polymerase chain reaction (RT-PCR; Fig. 1C) and found that most *REEP* PCR products were the expected size, suggesting that REEP5 and REEP6 might be endogenously expressed and involved in CXCR1 signaling, and that overexpression might enhance signaling. IL-8 triggered luciferase expression in a dose-dependent manner with maximal effects at approximately 100 nM. IL-8-dependent luciferase expression in exogenous REEP5 or REEP6-expressing cells was higher than expression in parent cells, whereas the EC_50_ values of IL-8 were fairly similar (Fig. 1D).

### REEP5 and REEP6 interact with CXCR1 but not CXCR2

To investigate the interaction of REEPs and CXCR1, cells expressing HA-tagged receptors and REEPs were applied for co-immunopreciptitation (Fig. 1E). Both REEPs co-precipitated with CXCR1 but not CXCR2, which could explain the reporter gene assay results. Western blots of total cell lysates revealed multiple bands, indicating that both receptors were likely glycosylated. The homology of CXCR1 and CXCR2 was relatively low in the N-terminal, second extracellular loop, and C-terminal regions; thus, chimeric CXCR1 and CXCR2 receptors were generated for binding experiments (Fig. 1F). Most chimeric receptors interacted with REEP6, but CXCR1 containing the CXCR2 N-terminal region did not bind REEP6, implying that the N-terminal region was required for the CXCR1 and REEP6 interaction (Fig. 1G).

### CXCR1-mediated signaling is down-regulated in cells lacking REEP5 and REEP6

**Figure 2:**
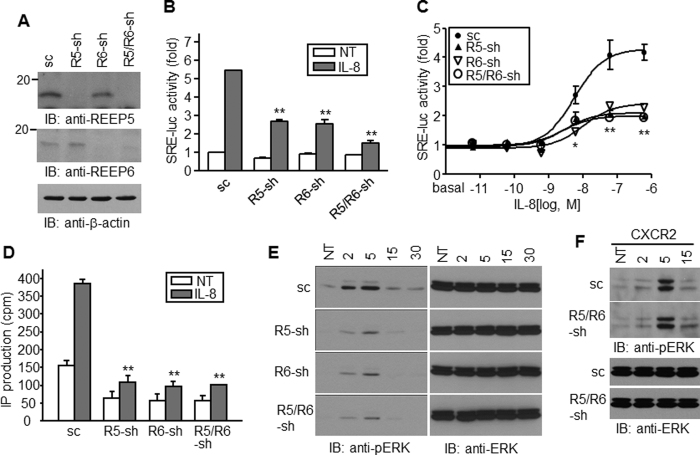
Depletion of REEP5 and REEP6 causes a decrease in CXCR1 signaling. (**A**) HEK293 cells were infected with lentivirus containing REEP5 and REEP6 shRNA, and the expression of the target proteins was determined by immunoblotting with anti-REEP5 or anti-REEP6 antibodies. (**B**) HEK293/Gα_15_ cells expressing shRNAs were transfected with *CXCR1* and *SRE*-*luc* reporter plasmid. Reporter gene assays were performed after treatment with 10 nM IL-8. Luciferase actitivies are presented by relative fold change to nontreated control. (**C**) Luciferase activities were determined in the cells treated with 10-fold serially diluted IL-8 (from 602 nM). (**D**) An IPs production assay was performed with HEK293 cells expressing REEP shRNAs and CXCR1. Cpm shows raw value of radioactivity. NT, no treatment. (**E** and **F**) HEK 293 cells expressing CXCR1 (**E**) or CXCR2 (**F**) were treated with IL-8 for the indicated times min, and analyzed by western blotting to determine ERK1/2 phosphorylation. sc, scrambled shRNA; sh, shRNA. *p < 0.05, **p < 0.01. All experiments were performed at least three times.

Western blotting with REEP5 and REEP6 antibodies revealed endogenous expression in HEK293 cells. To clarify their roles in CXCR1-mediated signaling, we constructed REEP5 and REEP6-specific shRNAs using a lentiviral system and infected HEK293 cells. After confirming the downregulation of each REEP, cells expressing REEP6-shRNA were re-infected with a lentivirus containing REEP5 shRNA to generate cells lacking both REEPs (Fig. 2A). The cells were transfected with *CXCR1* and *SRE*-*luc* in a 4:1 ratio and treated with IL-8 to examine receptor activation. The SRE-derived luciferase activities were significantly decreased in cells lacking REEP5 or/and REEP6 (Fig. 2B). IL-8 triggered luciferase activity in a dose-dependent manner, whereas potency and maximum efficacy were remarkably decreased in REEP5 and REEP6-deficient cells, implying that they were responsible for CXCR1-mediated signaling (Fig. 2C).

To explore early receptor signaling events, the activation of phospholipase C-β was determined by measuring production of inositol phosphates (IPs). IL-8 stimulated IPs production in cells expressing CXCR1. In cells lacking REEP5 and REEP6, the basal IPs level was lower, and IL-8-stimulated IPs production was negligible, indicating significantly downregulated signaling (Fig. 2D). Furthermore, IL-8 induced intracellular calcium release which was sustained for more than one minute in the control cells, whereas the calcium increase was shortly decreased in cells lacking REEP5 and REEP6 (see Supplementary Fig. S1). ERK phosphorylation occurred 2 min after IL-8 treatment, peaked at 5 min, and then decreased. Phosphorylation was detected in cells lacking REEP5 and REEP6, but at much lower levels than in control cells (Fig. 2E). In addition, absence of each protein led to downregulation of IL-8-induced cellular responses with similar extent to those in cells lacking both proteins. REEP5 and REEP6 are quite similar each other regarding to structure and they may function in a cooperative way. Immunoprecipitation assay performed with cells expressing both proteins revealed that they bind each other, suggesting that they functionally work together (see Supplementary Fig. S2). Conversely, IL-8 stimulated ERK phosphorylation in cells expressing CXCR2 regardless of REEP5 or REEP6 expression (Fig. 2F), suggesting that IL-8 barely elicited cellular signaling cascades through CXCR1 in the absence of REEP5 and REEP6.

### REEP5 and REEP6 do not influence membrane expression of CXCR1

**Figure 3:**
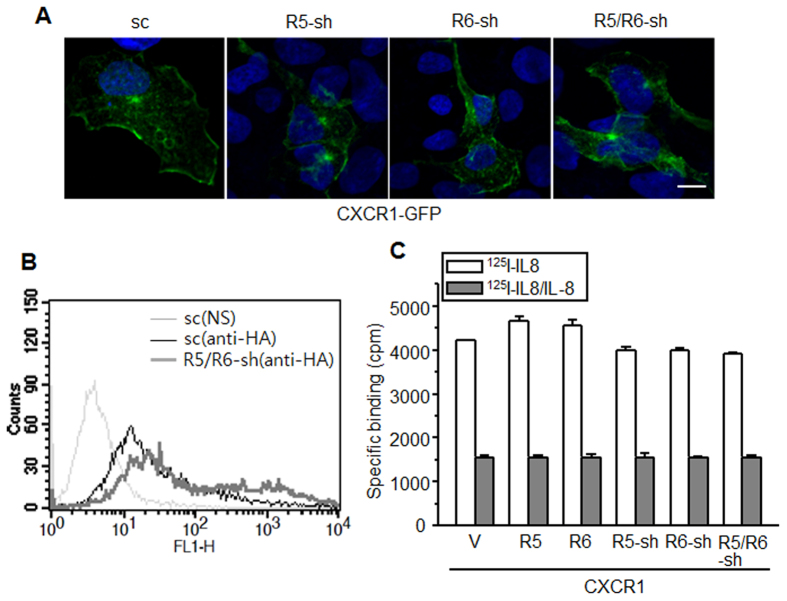
REEP5 and REEP6 might be unnecessary for membrane expression of CXCR1. (**A**) HEK293 cells lacking *REEP5* and *REEP6* were transfected with a GFP-tagged CXCR1 plasmid. Forty-eight hours post-transfection, the cells were fixed with 4% PFA and examined by confocal microscopy. The scale bar denotes 10 μm. (**B**) FACS analysis of CXCR1 expression. Membrane expression of HA-tagged CXCR1 in HEK293 cells expressing shRNAs was determined with anti-HA and FITC-conjugated secondary antibodies. Counts indicate relative cell number. NS: no staining. (**C**) A ligand binding assay using ^125^I-IL-8 was performed in HEK293 cells expressing CXCR1 in the presence of 200 nM unlabeled IL-8. Bonferroni’s Multiple Comparison Test for Vector versus the other groups revealed *p* > 0.05. The results are from at least three independent experiments.

To determine the effect of REEPs on CXCR1 membrane expression, genes encoding C-terminal GFP-tagged CXCR1 were introduced into cells. Fluorescence signals were enriched in cell margins regardless of REEP expression (Fig. 3A). To investigate receptor membrane expression, cells expressing HA-tagged CXCR1 with REEP5 and REEP6 shRNAs or scrambled shRNA were analyzed by FACS, and the results indicated that CXCR1 was expressed in the plasma membrane in the absence of REEPs (Fig. 3B). Furthermore, the antibody binding signals were slightly higher in the shRNA-containing cells compared with control cells, implying that dynamic expression of CXCR1 in plasma membrane may be downregulated or expression level become higher in cells lacking the REEPs regardless of ligand binding activity (see Fig. 3C).

To investigate the functional expression of the receptors, we performed ligand-binding assays using cells expressing REEPs or shRNAs with CXCR1. Radioactivity was slightly enhanced in REEP5 and REEP6-expressing cells, but slightly decreased in shRNA-expressing cells. However, the changes were not statistically significant (Fig. 3C), which implied that REEP5 and REEP6 might not affect receptor localization to the plasma membrane, but could influence the posttranslational modification of CXCR1. Nonetheless, the CXCR1 glycosylation patterns were similar among cells overexpressing or lacking REEPs, implying that REEPs might not affect CXCR1 glycosylation (data not shown).

### REEP5 and REEP6 might be involved in IL-8-stimulated internalization of CXCR1

**Figure 4:**
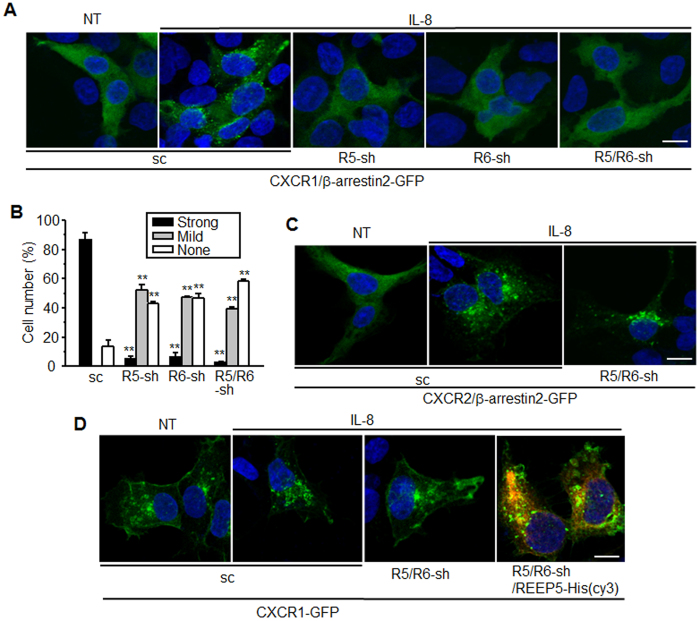
Depletion of REEP5 and REEP6 causes a defect in IL-8-stimulated internalization of CXCR1 and recruitment of β-arrestin2. (**A**) HEK293 cells containing shRNAs were transfected with CXCR1 and β-arrrestin2-GFP plasmids, treated with 10 nM IL-8 for 30 min, and fixed with 4% PFA. GFP signals were detected by confocal microscopy. (**B**) The bar graph presents the percentage of cells showing different internalization signals. The intensity was arbitrarily determined by brightness. The graph describes % of total cells. Supplementary Fig. S3 shows clustering patterns of β-arrestin2-GFP determined as strong, mild, and none. One way-ANOVA was performed between control group(sc) and REEP-knockdown cells(sh) for each clustering pattern of β-arrestin2-GFP. **p < 0.01. (**C**) IL-8-stimulated β-arrestin2 internalization in cells expressing CXCR2 was not affected by the depletion of REEP5 and REEP6. (**D**) HEK293 cells containing shRNAs were transfected with the CXCR1-GFP alone or CXCR1-GFP and REEP5-His plasmids. The cells were treated IL-8 for 30 min and applied for immunostaining with anti-His antibodies. GFP signals were detected by confocal microscopy. All images are representative of three independent experiments. NT, not treated; sc, scrambled shRNA; sh, shRNA. The scale bar denotes 10 μm. All experiments were performed at least three times.

Following transfection with *CXCR1* and *β*-*arrestin2*-*GFP*, diffuse GFP signals appeared in the cytosol and were prominently clustered following IL-8 treatment, indicating that β-arrestin2 mediates CXCR1 internalization stimulated by the ligand. However, the clusters were not detected in cells expressing the REEP5 and REEP6 shRNAs (Fig. 4A). Photographed cells were sorted into no, moderate, and strong change patterns. Most of the scrambled shRNA-expressing cells exhibited strong β-arrestin2-GFP clustering signals. However, the signals were barely detectible in the REEP shRNA-expressing cells. Nearly half of the cells showed no clustering signal and the other half exhibited weak or mild clustering signals in the presence of REEP shRNAs, which implied that REEP5 and REEP6 might be involved in receptor activation and internalization (Fig. 4B). When cells expressing CXCR2 and β-arrestin2-GFP were treated with IL-8, GFP clustering readily occurred irrespective of REEP shRNA expression, suggesting that REEP5 and REEP6 are unnecessary for CXCR2-mediated cellular responses (Fig. 4C). To confirm the results, *CXCR1*-*GFP* was introduced into cells expressing scrambled or REEP shRNAs. In the scrambled shRNA-expressing cells, GFP signals vanished from the plasma membrane and were clustered in the cytosol following IL-8 treatment, whereas no clusters were detected in REEP shRNA-expressing cells (Fig. 4D). To confirm the specific effect of REEP5 and REEP6 depletion on CXCR1 internalization, cells containing shRNAs for both REEPs were transfected with *REEP5* gene which has the sequences different from shRNA target site, and treated with IL-8. As shown in right panel of Fig. 4D, IL-8-stimulated CXCR1 internalization was recurred by REEP5 expression.

**Figure 5:**
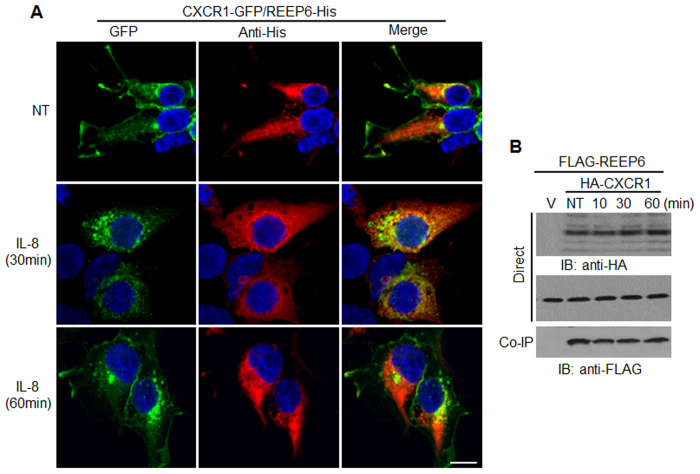
IL-8 treatment might not alter the interaction properties between REEP5, REEP6, and CXCR1. (**A**) HEK293 cells expressing *CXCR1*-*GFP* and His-tagged *REEP6* were treated with IL-8, fixed, and prepared for immunocytochemistry using anti-His antibodies and secondary Alexa 594-conjugated antibodies. Fluorescence signals were observed by confocal microscopy. The scale bar denotes 10 μm. (**B**) HEK293 cells expressing FLAG-REEP6 and HA-CXCR1 were treated with IL-8 for the indicated times and subjected to immunoprecipitation with anti-HA antibodies. Co-precipitated REEP6 was detected by western blotting with anti-FLAG antibodies. The results are from at least three independent experiments.

To investigate the subcellular localization of REEP and CXCR1, both were introduced into HEK293 cells. CXCR1-GFP mainly localized in the plasma membrane, whereas REEP5 and REEP6 existed in the cytosol. IL-8 treatment induced intracellular clustering of CXCR1-GFP and the fluorescent signals partially overlapped with those of REEPs, suggesting that REEPs might be involved in CXCR1 translocation (Fig. 5A). REEP6 signals were dispersed in the cytosol and were not changed by IL-8.

During IL-8-stimulated CXCR1 activation and internalization, the interaction between CXCR1 and REEPs could be altered; thus, an immunoprecipitation assay was performed with cell lysates harvested at different times. Western blots revealed that the amount of co-precipitated REEP6 remained the same over time (Fig. 5B), which implied that although dynamic changes might occur, the overall interaction of REEP and CXCR1 was unchanged.

### CXCR1-stimulated cellular responses are downregulated in lung cancer cells lacking REEP5 and REEP6

**Figure 6:**
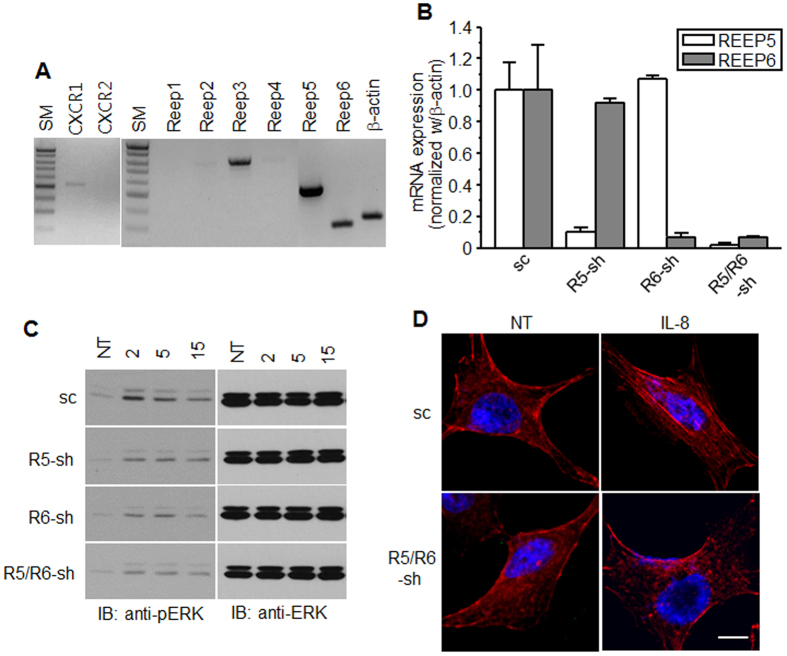
CXCR1 signaling in A549 cell lines was decreased by the depletion of REEP5 and REEP6. (**A**) Using RNAs from A549 cells, RT-PCR was performed with specific CXCR1, CXCR2, and REEP1–6 primers. The PCR products were resolved on a 1% agarose gel. SM, size markers (1 Kb Plus DNA ladder from Invitrogen). (**B**) qRT-PCR was performed for REEP5 and REEP6 mRNA in A549 cells infected with lentivirus containing shRNAs. Relative mRNA levels of REEP5 and REEP6 were normalized with β-actin mRNA and are shown as mean ± S.E (n = 4). The bar graphs are presented by relative value to sc control. (**C**) A549 cells containing shRNAs were treated with IL-8 for the indicated times (min) and analyzed by western blotting with anti-pERK1/2 antibodies. NT, not treated. (**D**) A549 cells containing shRNAs were treated with IL-8 for 20 min, fixed, and stained with phalloidin-TRITC. The scale bar denotes 10 μm. The results are from at least three independent experiments.

A549 lung cancer cells were used to evaluate the roles of REEPs in IL-8-dependent cellular responses. RT-PCR confirmed CXCR1 expression and revealed that REEP5 and REEP6 are likely dominant REEPs in A549 cells (Fig. 6A). To explore the role of REEPs in cellular responses mediated by endogenously expressed CXCR1, A549 cells lacking REEP5 and REEP6 were established using a lentivirus containing shRNA (Fig. 6B). IL-8 treatment increased ERK phosphorylation, which was significantly downregulated in cells lacking REEP (Fig. 6C). However, phosphorylation still occurred in the absence of REEPs, indicating that REEPs are not solely responsible for CXCR1-mediated ERK phosphorylation. IL-8 also induced actin polymerization in A549 cells, which was not detected in cells lacking REEP, suggesting that the migration signal might be blocked (Fig. 6D).

**Figure 7:**
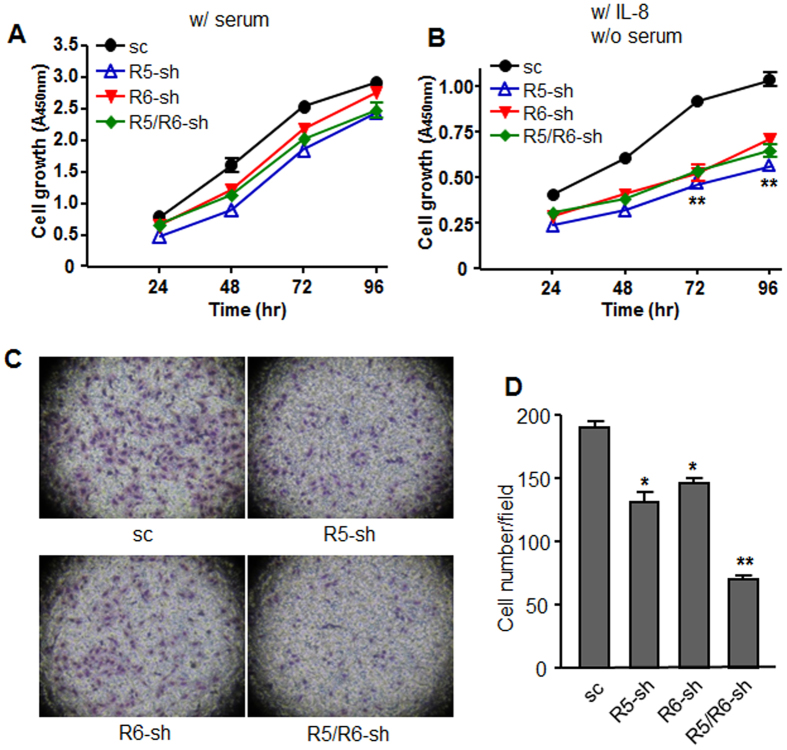
Depletion of REEP5 and REEP6 weakens the proliferation and invasion abilities of A549 cells. A549 cells containing shRNAs were seeded in 96-well plates (2,000/well), and cultured with (**A**) medium containing 10% FBS or (**B**) serum-free medium containing 100 ng/ml of IL-8. At 24 h, 48 h, 72 h, and 96 h after treatment, the cells were incubated with a CCK-8 solution for 2 h, and a microplate reader was used to measure absorbance at 450 nm. (**C**) A549 cells (5 × 10^4^) were seeded in the upper chamber of a Transwell^®^ plate and treated with 100 ng/ml of IL-8. Medium containing 10% FBS was added to the lower well. Eighteen hours later, the Transwell^®^ inserts were removed and stained with hematoxylin. (**D**) The cells that migrated were counted under 200x magnification in five microscope fields. The average number of cells that migrated per field is shown in the graph. *p < 0.05; **p < 0.01. The results are from at least three independent experiments.

A proliferation assay revealed that REEP-deficient cells grew slowly in the presence of 10% FBS compared to scramble shRNA-expressing cells, but the differences were not statistically significant (Fig. 7A). To explore the effect of IL-8, cells were cultured without serum and treated with IL-8 daily (Fig. 7B). The cells grew in the absence of serum, but the growth rate was significantly low (*p* < 0.01). The cells lacking REEP did not grow well, suggesting that REEP5 and REEP6 might be involved in CXCR1-mediated growth.

Despite its chemotactic activity, IL-8 barely induced the migration of A549 cells. When serum was used as a chemoattractant, only a few A549 cells migrated (data not shown). When cells were treated with IL-8 and medium containing 10% FBS, many cells harboring control shRNAs migrated, but the migration efficiency was downregulated by REEP5 and REEP6 shRNA expression. Furthermore, expression of both shRNA prominently decreased cell migration (Fig. 7C and D).

Because IL-8-treated A549 cells are highly chemotactic, IL-8 likely induces pro-metastatic genes. To determine effects at the mRNA level, real-time quantitative RT-PCR (qRT-PCR) analysis was performed using cells treated with IL-8. The expression of urokinase-type plasminogen activator (*uPA*), its cognate receptor (*uPAR*), matrix metalloproteinase-2 (*MMP2*), and *MMP9* were induced by IL-8, and IL-8 and IL-6 expression was increased (see Supplementary Fig. S4), indicating that IL-8 might cause cells to acquire a motile and proliferative phenotype by stimulating gene expression. However, gene induction was abolished in cells lacking REEP5 and REEP6, implying that these REEPs play a pivotal role in CXCR1-mediated gene expression.

### Deficiency in REEP5 and REEP6 inhibits growth and metastasis of lung cancer cells

**Figure 8:**
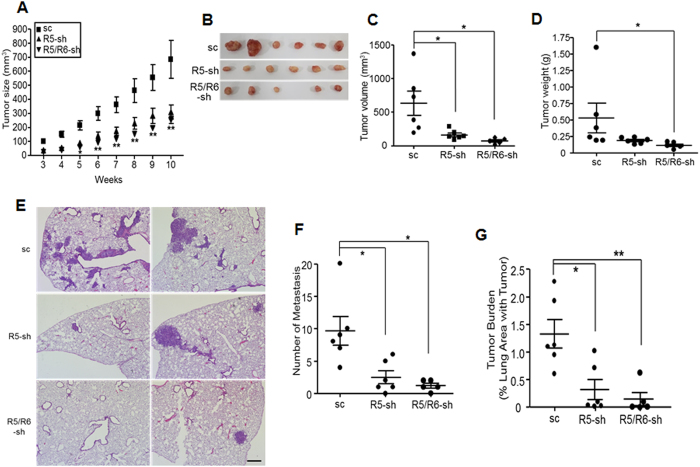
The tumorigenesis and lung metastasis of A549 cells is suppressed by the silencing of *REEP5* and *REEP6*. (**A**) A549 cells containing shRNAs were injected into the flank of NOD/SCID mice. Tumor volumes were measured every week and calculated using the following formula: 0.5 × length × width^2^ (mm^3^) (n = 6 mice per group, n = 5 of the REEP5 and REEP6-shRNA groups due to accidental death during the experiment). **Statistical significance of the difference between the REEP5 and REEP5/6 shRNA groups from controls; *p* < 0.01. (**B**) Ten weeks after implantation, the tumors were resected for analysis. The pictures show the tumors from each group. The volume (**C**) and weight (**D**) of each tumor were measured and presented graphically. The results are expressed as mean ± S.E.; *p < 0.05. (**E**) The mice were sacrificed 14 weeks after injection and lung sections were examined by hematoxylin/eosin staining. Pictures are representative of lung section data. Scale bar, 200 μm. (**F**) The tumor nodules in lungs of mice were counted. (**G**) The metastatic ability was represented as tumor burden. Results are expressed as mean ± S.E.; *p < 0.05; **p < 0.01.

To investigate the effect of REEPs on lung tumor growth and metastasis *in vivo*, A549 cells containing scrambled shRNA, or REEP5 and REEP6 shRNAs, were injected into NOD/SCID mice to generate a primary tumor. The tumor growth was significantly different between control and shRNA-containing groups (Fig. 8A), but body weights were similar in all groups (data not shown). After surgical resection, the determination of tumor volume and weight revealed that shRNA-containing groups were significantly different from controls, although errors were apparent in the weekly measurements compared to these results (Fig. 8B–D). A549 cells infected with a lentivirus carrying shRNAs were used for experiments without cloning; nevertheless, tumor size from each mouse in the shRNA-containing groups was similar, although the size varied in the control group, suggesting that REEP deficiency might influence consistent cellular responses. Statistical analysis revealed that tumor growth was remarkably reduced in the mice lacking both REEP5 and REEP6 (Fig. 8D).

Histological analysis of lungs from the mice revealed that the A549 cells produced a large number of lung nodules and metastatic tumor burden in control mice, whereas both events were markedly decreased in the mice injected with cells lacking REEP5, or REEP5 and REEP6 (Fig. 8E–G), suggesting that REEP5 and REEP6 might influence lung cancer cell metastasis.

## Discussion

The heterologous expression of GPCRs highlights the importance of specific accessory proteins that contribute to the folding and trafficking of GPCRs to the plasma membrane^15^. Among these, REEPs appear to assist the cell surface expression of olfactory receptors that are scarcely expressed in heterologous transfection systems^14^, indicating that REEPs might fine-tune the expression and functional activation of GPCRs in a protein-specific manner. Although many studies have focused on signaling events in neuronal cells, REEPs are also expressed in other tissues and cells as shown in the results of this study and others^16^,^17^. Therefore, defining the role of REEPs in the functional expression of various GPCRs may help to understand various physiological activities of GPCRs.

In this study, REEP5 and REEP6 played essential roles in the IL-8-stimulated activation of CXCR1, but not CXCR2. The results revealed that REEP5 and REEP6 interacted with the extracellular N-terminal regions of CXCR1, but not CXCR2, suggesting that REEPs are CXCR1-specific binding partners. The overexpression of REEP5 and REEP6 cellular responses by IL-8-stimulated CXCR1 and the downregulation of REEP expression using shRNAs led to the downregulation of signaling events, including ERK phosphorylation, PLC-β activation, and intracellular calcium release. Interestingly, the downregulation effects on signaling pathways in cells lacking either REEP5 or REEP6 were nearly the same or slightly lower compared to those in cells lacking both REEPs. Because REEP5 and REEP6 are similar in primary structure, compensation for the lack of one isotype could be expected. However, our results suggested that there must be critical amount of the proteins necessary to induce downstream signaling. Nevertheless, migration assays and *in vivo* experiments revealed that a deficiency of both proteins generated more profound effects compared to the lack of either protein, implying that the cellular responses induced by the proteins occurred in a dose-dependent manner.

REEP1 facilitates the membrane expression of specific GPCRs^14^,^18^. However, CXCR1 readily localized to the plasma membrane in the absence of both REEP5 and REEP6. Further, FACS analysis revealed a stronger membrane concentration of CXCR1 in cells lacking REEPs compared to control cells, suggesting that REEP5 and REEP6 function differently than other accessory proteins and enhance membrane expression of GPCRs. CXCR1 tends to be internalized by IL-8 treatment in a β-arrestin-dependent manner^19^. Our results confirmed that internalized CXCR1 overlapped with the early endosome marker EEA1 (data not shown), whereas IL-8-dependent internalization of β-arrestin and CXCR1 were abrogated in the absence of REEP5 and REEP6, implying that REEP5 and REEP6 were involved in receptor internalization rather than in membrane expression and ligand binding. In fact, prior reports have indicated that internalization of receptors might be a prerequisite for the amplification of signaling events^20^,^21^. For this reason, the activities of intracellular signaling molecules were likely downregulated in cells lacking REEP5 and REEP6. However, the mechanisms underlying REEP5 and REEP6 regulation of CXCR1 endocytosis are still unclear.

IL-8 is a multifunctional chemokine secreted by numerous tumor cells and cancer-associated stromal cells including leukocytes, fibroblasts, and endothelial cells^22^,^23^. Likewise, IL-8/CXCR1 signaling is elevated by the tumor microenvironment and promotes tumor progression via activation of the signaling pathways that enhance tumor cell proliferation, migration, and angiogenesis, and by recruiting macrophages and neutrophils to the tumor site^24^,^25^,^26^. Gene expression analysis revealed that REEP5 was overexpressed in some advanced cancer tissue, whereas its roles in cancer progression were not clarified yet^16^. To verify the effect of REEP5 and REEP6 on CXCR1-mediated cancer cell responses, A549 lung cancer cells were used in this study because the cells only express CXCR1^27^. The results clearly indicated that REEP5 and REEP6 were required for proper growth and migration of A549 lung cancer cells *in vitro* and *in vivo*. Furthermore, metastasis from the primary tumor to the lung was considerably reduced by REEP5 and REEP6. Because REEP5 and REEP6 could be involved in other regulatory mechanisms, the xenograft model results cannot fully explain the direct effects of REEP deficiency on CXCR1-mediated cellular responses. Nevertheless, the regulation of CXCR1 signaling by REEPs likely influenced the growth and metastasis of the lung cancer cells.

Taken together, these data demonstrated that REEP5 and REEP6 play essential roles in CXCR1-mediated cellular responses by direct interaction with the receptor. Previous reports have shown that the accessory proteins are responsible for cell surface expression of GPCRs^28^,^29^,^30^. However, these results revealed that REEP5 and REEP6 could somehow enhance CXCR1-stimulated cellular responses by inducing endocytosis of the receptors after ligand binding, rather than membrane expression. Thus, REEP5 and REEP6 could be novel regulators of GPCR signaling whose functional mechanisms differ from other accessory proteins.

## Materials and Methods

### Materials

Human recombinant IL-8 was purchased from PeproTech (Rocky Hill, NJ). The following primary antibodies were used: anti-His antibodies (ab18184, Abcam, Cambridge, UK); anti-REEP5 (sc-133405), anti-REEP6 (sc-133410), anti-pERK (sc-7383), anti-ERK antibodies (sc-94), anti-β-actin (sc-47778) (Santa Cruz Biotechnology, Santa Cruz, CA); anti-hemagglutinin (HA)(H3663) and anti-FLAG antibodies (F3165); phalloidin-TRITC (P1951, Sigma-Aldrich, St. Louis, MO). All reagents were from Sigma-Aldrich unless otherwise stated. Molecular weight marker (DokDo-MARK^TM^, broad range) in SDS-PAGE and DNA size ladder marker (1 Kbp plus 100 bp) were from Invitrogen(Carlsbad, CA).

### Cell culture

HEK293, HEK293T, and A549 cells (American Type Culture Collection, Manassas, VA) were cultured in DMEM or RPMI1640 (Welgene, Daegu, Korea) containing 10% fetal bovine serum (FBS), 1% penicillin (100 IU/ml), and 100 μg/ml streptomycin (Invitrogen, Carlsbad, CA).

### Plasmid constructs

The cDNA of *CXCR1* and *CXCR2* were generated by PCR using genomic DNA from HEK293 cells, cloned into the pcDNA3.1 vector (Invitrogen, Carlsbad, CA), and verified by sequencing. They were also subcloned into the pcDNA3.1HA. For chimeric *CXCR1* and *CXCR2*, individual cDNA fragments corresponding to the 5′- or 3′- end of the receptor cDNAs or to the region of overlap between the two receptors were amplified by PCR. Each *CXCR1* and *CXCR2* fragment was subjected to a second round of PCR to generate the chimeric cDNAs, which were inserted into pcDNA3.1HA and verified by sequencing. Human *REEP* (21C Frontier Human Gene Bank, Daejeon, Korea) was subcloned into the pcDNA3.1 or pcDNA3.1-mychis vector. CXCR1 and β-arrestin2 genes were inserted into pEGFP-N1.

### Lentivirus generation and establishment of REEP-deficient cells

Lentiviral vectors containing shRNAs were used to downregulate the expression of each REEP (Thermo Scientific Inc., Waltham, MA) following transfection into HEK293 cells with pcDNA3.1mychis/REEP plasmids. The shRNA constructs were inserted into the lentiviral FG12 vector and transfected into HEK293T cells with accessory plasmids using a calcium phosphate precipitation method. Two days later, the culture supernatants were harvested and concentrated using Amicon Ultra columns (Millipore, Billerica, MA). An EGFP-expressing lentivirus served as a control to evaluate infection efficiency. The multiplicity of infection was 10, and the expression of target proteins was determined by western blotting.

### RT-PCR and qRT-PCR

RNA was extracted from cells using TRIzol^®^ (Invitrogen) and reverse transcription was performed with 2 μg of total RNA and SuperScript™ II Reverse Transcriptase according to the manufacturer’s instructions (see Supplementary Table 1). Following PCR amplification (30 cycles), PCR products were loaded on a 1.5% agarose gel. The iQ^TM^ SYBR Green Supermixture and an iCycler PCR thermocycler (Bio-Rad, Hercules, CA) with gene-specific primers (Beacon Designer 2.1, Biosoft International, Palo Alto, CA) were used for qRT-PCR. The mRNA levels were normalized to glyceraldehyde-3-phosphate dehydrogenase. Data from three independent experiments carried out in triplicate are presented as the mean ± S.E.

### Immunoprecipitation and western blotting

A day before transfection, HEK293 cells were plated in 60 mm dishes. The HA-tagged CXCRs and REEP plasmids were co-transfected with Lipofectamine^®^ 2000 (Invitrogen) according to the manufacturer’s instructions. After 36 h, the cells were washed with ice-cold phosphate-buffered saline (PBS) and solubilized with lysis buffer containing 50 mM Tris-HCl (pH7.5), 150 mM NaCl, 1% Triton™ X-100, and protease inhibitor cocktail for 30 min on ice. The lysates were centrifuged at 15,000 rpm for 15 min at 4 °C and incubated with anti-HA antibody-conjugated beads at 4 °C for 2 h. The beads were washed four times with lysis buffer, and the bound proteins were eluted by boiling in sodium dodecyl sulfate (SDS) buffer and separated by SDS-polyacrylamide gel electrophoresis. To examine protein expression, clarified lysates (20 μg) were electrophoresed, transferred onto a nitrocellulose membrane, probed with antibody, and detected using an ECL assay kit (GE Healthcare Life Science, Pittsburgh, PA).

### Reporter gene assays

HEK293 cells stably expressing Gα_15_ were cultured in 24- or 48-well plates and transfected with CXCR1, REEPs, or pGL3/SRE-luciferase reporter gene in a 4:4:1 ratio. The cells were washed and provided serum-free media, then treated 24 h later with 10 nM IL-8 (R&D Systems, Minneapolis, MN) for 6 h, washed with PBS, and solubilized with lysis buffer. The cell extract luciferase activity was determined using a standard luciferase assay (BioTek Instrument, Inc., Winooski, VT). All data were from at least three independent experiments and normalized to untreated groups.

### Ligand binding assay

IL-8 was radioiodinated using the chloramine-T method and purified by chromatography on a Sephadex G-50 column (Sigma-Aldrich) in 0.01 M acetic acid and 0.1% bovine serum albumin (BSA). HEK293 cells were transfected with CXCR1 (300 ng of DNA/well in 12-well plates) with Effectene^®^ (Qiagen, Hilden, Germany). Forty-eight hours later, the cells were washed and incubated for 1 h with binding buffer (serum-free DMEM with 0.1% BSA, pH 7.4) containing 100,000 cpm ^125^I-labeled IL-8 in the presence of 200 nM of cold ligand. The cells were washed twice with ice-cold PBS. Cell lysates were resolved in 1% SDS and 0.2 M NaOH and radioactivity was determined using a Wallac 1489 Wizard 3 γ-counter (PerkinElmer Inc., Waltham, MA).

### Inositol phosphates (IP)-generation assay

HEK293 cells containing shRNAs were seeded in a 12-well plate and transfected with CXCR1 plasmids. The cells were incubated in M199 medium in the presence of 1 μCi/ml/well *myo*-[3H] inositol (Amersham Biosciences) for 20 h. Afterward, the medium was removed and the cells were washed with 0.5 ml Buffer A (140 mM NaCl, 20 mM HEPES, 4 mM KCl, 8 mM D-glucose, 1 mM MgCl_2_, 1 mM CaCl_2_, and 1 mg/ml fatty acid-free BSA). Cells were then preincubated for 30 min with Buffer A containing 10 mM LiCl followed by the addition of IL-8 at 37 °C for 30 min. The reaction was terminated with 0.5 ml of ice-cold 10 mM formic acid. After 30 min, the extracts were transferred to columns containing Dowex™ anion-exchange resin (AG-1-X8 resin, Bio-Rad). Total IPs were eluted with 1 ml of ammonium formate and 0.1 M formic acid. Radioactivity was determined using a Tri-Carb^®^ 3100TR scintillation counter (PerkinElmer Inc.).

### Fluorescence assay

To examine the subcellular localization of the proteins, the plasmids were introduced into HEK293 cells or REEP-knockdown HEK293 cells cultured on cover-glass. Forty-eight hours later, cells were fixed with 4% paraformaldehyde (PFA) and GFP-tagged protein localization was investigated by confocal microscopy. Internalization of receptor or β-arrestin2 was determined after 10 nM IL-8 treatment for 30 min. Fixed cells were incubated with primary antibodies (anti-His antibody) followed by TRITC-labeled secondary antibodies to detect exogenous REEP and early endosome expression. To visualize the actin cytoskeleton, the cells were fixed for 30 min in 4% paraformaldehyde and incubated with phalloidin-TRITC for 30 min. To stain the nucleus, cover glasses were incubated with Hoechst33342 solution.

### Proliferation assay

Cell growth was evaluated by seeding A549 cells containing shRNAs in 96-well plates (2 × 10^3^/well). After 24 h, the cells were cultured with 10% FBS-containing media or treated with IL-8 in serum-free media. Cell growth was determined by using cell counting kit-8 (CCK-8) (Dojindo Molecular Technologies, Kumamoto, Japan). The absorbance was measured at 450 nm using a microplate reader.

### Invasion assay

Transwell^®^ inserts with 8-mm pores (Corning Inc., Corning, NY) were coated with Matrigel (Invitrogen, Carlsbad, CA) for 2 h at 37 °C. A549 cells (5 × 10^4^) in RPMI media containing 0.1% BSA and 100 ng/ml IL-8 were added to the upper wells. Medium containing 10% FBS was added to the bottom wells and the plates were incubated in a 5% CO_2_ humidified incubator at 37 °C for 18 h. Transwell^®^ inserts were washed in PBS and non-migrated cells were removed with a cotton swab. The membranes were fixed in 4% PFA and stained with hematoxylin. Migrated cells were counted by light microscopy.

### Xenograft model

The animal experiments were approved by the Korea University Institutional Animal Care & Use Committee (KUIACUC-2013-239) and carried out in accordance with the relevant guideline and regulation. A549 cells (2 × 10^6^ cells/spot) were injected subcutaneously into the right flank of 6-week-old female NOD/SCID mice (KOATECH, Pyeongtek, Korea). Tumor growth was monitored weekly using electronic calipers: tumor volume = 0.5 × length × width^2^. When tumor volumes reached approximately 1000 mm^3^, tumors were surgically resected and weighed. The lung tissues harvested at the experiment end were fixed in 10% buffered formalin for 24 h and processed for histological analysis. Sections (5 μm) were mounted on glass slides and stained with hematoxylin and eosin. All metastatic tumor nodules were counted and nodule sizes were measured by light microscopy.

### Statistical analyses

Data were analyzed by nonlinear regression with a sigmoidal dose response. The IL-8 concentrations that induced half-maximal stimulation (EC_50_) were calculated using GraphPad PRISM4 software (GraphPad Software Inc., San Diego, CA). Group means were compared using Student’s *t*-test or one-way analysis of variance (ANOVA) followed by Bonferroni’s multiple comparison tests. All data are presented as mean ± S.E. of at least three independent experiments.

## Additional Information

**How to cite this article**: Park, C. R. *et al*. The accessory proteins REEP5 and REEP6 refine CXCR1-mediated cellular responses and lung cancer progression. *Sci. Rep.* **6**, 39041; doi: 10.1038/srep39041 (2016).

**Publisher's note:** Springer Nature remains neutral with regard to jurisdictional claims in published maps and institutional affiliations.

## Supplementary Material

Supplementary Information
