# Supplementary material for: The accessory proteins REEP5 and REEP6 refine CXCR1-mediated cellular responses and lung cancer progression
Source: Sci Rep. 2016 Dec 14;6:39041. doi: 10.1038/srep39041 (PMC5155276; doi:10.1038/srep39041)

The accessory proteins REEP5 and REEP6 refine CXCR1-mediated cellular responses and lung cancer progression

Cho Rong Park[1], Dong-Joo You[1], Sumi Park[1], Sunam Mander[1], Da-Eun Jang[2], Su-Cheong Yeom[2], Seong-Hyun Oh[3], Curie Ahn[4], Sang Heon Lee[5], Jae Young Seong[1], and Jong-Ik Hwang[1]*

[1]Graduate School of Medicine, [5]Department of Physical Medicine and rehabilitation, Korea University, Seongbuk-gu, Seoul 02841, Republic of Korea

[2]Graduate School of International Agricultural Technology, Seoul National University, 1447 Pyeongchang-Ro, Daewha, Pyeongchang, Kangwon, 25354, Republic of Korea

[3]College of Pharmacy, Gachon University, Incheon, 406-840, Republic of Korea

[4]Transplantation Research Institute, Cancer Research Institute, Seoul National University, Yongun-dong, Jongno-gu, Seoul 110-799, Republic of Korea

Supplementary Table 1. Primer sequences used for RT-PCR and real-time PCR

Primer	Sequence (5'-3')
Human CXCR1	F- CCTTCTTCCTTTTCCGCCAG
	R- AAGTGTAGGAGGTAACACGATG
Human CXCR2	F- ATTCTGGGCATCCTTCACAG
	R- TGCACTTAGGCAGGAGGTCT
Human REEP1	F- ACCATGGTGTCATGGATCATCTC
	R- CTAGGCGGTGCCTGAGCTGC
Human REEP2	F- TCAAGCTGAGTCGCCCCCGCCAG
	R- GACCATGGTGTCCTGGATGATCTC
Human REEP3	F- GACCATGGTGTCCTGGATGATCTC
	R- CTAAAAATACACTTGTGGTCG
Human REEP4	F- ACCATGGTGTCCTGGATGATCTG
	R- CTAGCTGTCCACGTCTGAG
Human REEP5	F- ACCATGTCTGCGGCCATGAGG
	R- TTAGGTGCTCTTCTTTTCTTC
Human REEP6	F- GAGCACTTCCTGGAGCAAAG
	R- AAGGGAACCAGGACAGGAGT
Human uPA	F- TTGCTCACCACAACGACATT
	R- GGCAGGCAGATGGTCTGTAT
Human uPAR	F- CCTCTGCAGGACCACGAT
	R- TGGTCTTCTCTGAGTGGGTACA
Human IL-6	F- GGTACATCCTCGACGGCATCT
	R- GTGCCTCTTTGCTGCTTTCAC
Human MMP2	F- CGGAAAAGATTGATGCGGTA
	R- TGCTGGCTGAGTAGATCCAG
Human MMP9	F- ATCCGGCACCTCTATGGTC
	R- CTGAGGGGTGGACAGTGG
Human IL-8	F- GAGCACTCCATAAGGCACAAA
	R- GGTTCCTTCCGGTGGT
Human GAPDH	F- CTCTGCTCCTCCTGTTCGAC
	R- AATCCGTTGACTCCGACCTT
Human β-actin	F- GATCAGCAAGCAGGAGTATGAC
	R- ATGGCAAGGGACTTCCTGTAAC

Supplementary method

Ca^{2+} Mobilization Assay

HEK293 cells were transiently transfected with CXCR1 plasmid and grown on poly-L-lysine coated glass coverslips for 24–48 h. Cells were then incubated in a physiological solution (138 mM NaCl, 6 mM KCl, 1 mM $MgSO_4$, 2 mM CaCl2, 1 mM Na_2HPO_4, 5 mM $NaHCO_3$, 5 mM glucose, 10 mM HEPES, and 0.1 % bovine serum albumin) with 5 μM fura-2/AM (Molecular Probes, Eugene, OR) at room temperature for 45 min. Cells were washed twice with the dye-free physiological solution to

remove extracellular dye and cleave any remaining intracellular AM-ester dye forms and mounted in an experimental chamber. The fluorescence intensity was followed kinetically after addition of IL-8 (100 ng/ml) using an IX71 fluorescence microscope (Olympus, Tokyo, Japan) coupled to a digital cooled CCD camera (CoolSNAP fx CCD camera, Roper Scientific, Tucson, AZ). Concentration–response curves were determined using GraphPad Prism 4 (GraphPad Software).

Supplementary Fig. S1. IL-8-stimulated intracellular calcium release was decreased in cells lacking REEP5 and/or REEP6. Arrowhead indicates IL-8 treatment

Supplementary Fig. S2. REEP5 and REEP6 bind each other.

Supplementary Fig. S3. Internalization and clustering patterns of β-arrestin2-GFP by IL-8 in HEK293 cells. Fig.4B graphs were developed by these patterns.

Supplementary Fig. S4. IL-8-stimulated metastatic gene expression is downregulated in the absence of REEP5 and REEP6. A549 cells containing shRNAs were incubated with (gray bars) or without (white bars) 10 nM IL-8 for 24 h. cDNAs were synthesized by reverse transcription, and RT-PCR was carried out using gene-specific primers. The relative amount of RNA obtained from each sample was calculated from a standard curve and normalized with *GAPDH* RNA. The bars presented by relative values to sc control. Data from three independent experiments performed in triplicate were combined and are presented as the mean±S.E. $*p < 0.05$; $**p < 0.01$.

Supplementary Fig. S1 Intracellular calcium release by IL-8

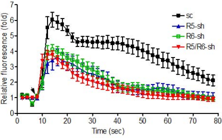

Supplementary Fig. S2 REEP5 and REEP6 bind each other

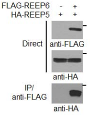

Supplementary Fig. S3 Localization patterns of β-arrestin2-GFP by IL-8 in cells expressing CXCR1 and shRNAs of REEP5/6

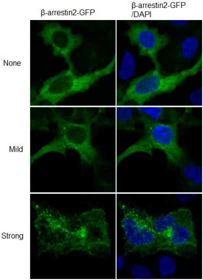

Supplementary Fig. S4 Analysis of gene expression by quantitative RT-PCR

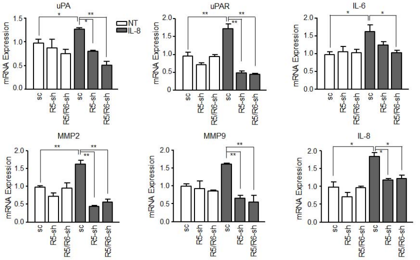

Supplement: Supplementary Information [file srep39041-s1.pdf]
